# Synthesis of 3,4-Dibenzyltetrahydrofuran Lignans (9,9′-Epoxylignanes)

**DOI:** 10.3390/molecules181113124

**Published:** 2013-10-24

**Authors:** Monika Pohjoispää, Kristiina Wähälä

**Affiliations:** Laboratory of Organic Chemistry, Department of Chemistry, P.O. Box 55, FIN-00014, University of Helsinki, Helsinki, 00560, Finland

**Keywords:** lignan, *trans*-3,4-dibenzyltetrahydrofuran, tetrahydrofuran, 9,9'-epoxylignane, synthesis

## Abstract

Different strategies for the racemic or enantiospecific total syntheses of plant and mammalian 3,4-dibenzyltetrahydrofuran lignans are reviewed and compared. The multi-step approaches have various key step strategies: Diels–Alder reactions, Stobbe condensations, Michael additions, alkylations, nitrile oxide cycloadditions, radical cyclisations, dianion and oxidative couplings.

## 1. Introduction

Phytoestrogens are plant derived compounds that may have estrogen-like actions in humans and animals. Lignans, together with the other main phytoestrogens—isoflavones, flavonoids and coumestans—are polyphenols and have structural similarity to the natural and synthetic steroid estrogens. Thus, depending on their concentration and other factors, they can act either like weak estrogens by binding to the estrogen receptors on cell membranes, or as estrogen antagonists by preventing estrogens from binding to the receptors [1].

**Figure 1:**
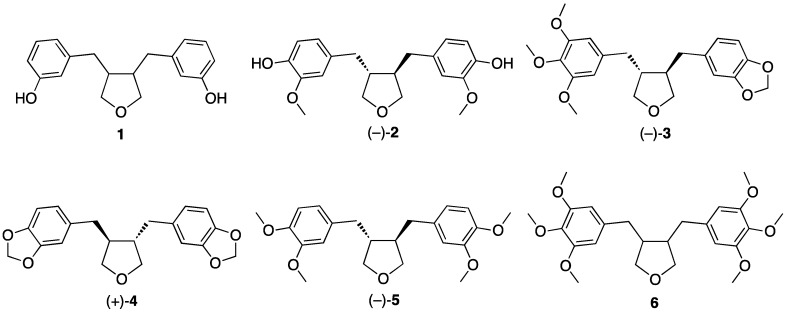
Naturally occurring 3,4-dibenzyltetrahydrofuran lignans (IUPAC names in parentheses). Mammalian lignan enterofuran (**1**, 3,3′-dihydroxy-9,9′-epoxylignane), and plant lignans anhydrosecoisolariciresinol aka shonanin (**2**, 4,4′-dihydroxy-3,3′-dimethoxy-9,9′-epoxylignane) [14,18,19], burseran (**3**, 3,4,5-trimethoxy-3′,4′-methylenedioxy-9,9′-epoxylignane) [12], dehydroxycubebin (**4**, (3,4),(3′,4′)-dimethylenedioxy-9,9′-epoxy-lignane) [20], brassilignan (**5**, 3,3′,4,4′-tetramethoxy-9,9′-epoxylignane) [21], and **6** (3,3′,4,4′,5,5′-hexamethoxy-9,9′-epoxylignane) [22] (there is some confusion however concerning the structure of compound **6**: Fuzzatti *et al*. have measured a positive value of the optical rotation for the isolated *trans*-3,4-dibenzyltetrahydrofuran lignan, but have drawn the structure as that of the (8*R*,8′*R*)-enantiomer [22], which in all the other reported cases is observed to have a negative value).

After the first mammalian lignans enterolactone and enterodiol, intestinal metabolites of plant lignans, were reported at the beginning of 1980s [2,3], the relevance of lignans for human health has been under active and extensive study. In the recent years new mammalian lignans have been found and identified or tentatively identified, e.g., *trans*-3,4-dibenzyltetrahydrofuran type lignans (9,9'-epoxylignanes) enterofuran (**1**) and anhydrosecoisolariciresinol (**2**) (Figure 1) [4,5,6,7].

Tetrahydrofuran-type lignans found in plants (Figure 1) are used both in Japanese folk medicine and in traditional Chinese medicine [8]. They have shown antioxidant [9], antibacterial [10] and insecticidal [11] activity and tumor-inhibitory properties [12]. Recent studies, in animals and humans, have suggested that a high consumption of phytoestrogens may reduce the risk of breast cancer. It is postulated that phytoestrogens may affect cancer incidence by altering production and metabolism of steroid hormones and their action at the cellular level [13]. Both the mammalian lignan enterofuran (**1**) and especially the plant lignan anhydrosecoisolariciresinol ((−)-**2**) have shown high affinity to human sex hormone binding globulin (SHBG) [14,15], which is the major plasma sex hormone transport protein with a high affinity to bind androgens and estrogens. (−)-**2** was able to completely prevent dihydrotestosterone from binding to the SHBG under the conditions tested.

Since the possible role of lignans and their metabolites in preventing hormone-dependent diseases has been recognized [16], synthetic methods for authentic standards as well as isotopically labelled analogues of these compounds are required [17]. Several routes for the synthesis of both racemic and chiral 3,4-dibenzyltetrahydrofuran lignan natural products have been reported in the literature. The majority of these procedures use Michael additions, dehydration of substituted 1,4-diols, or alkylation methodologies. Most of the reported syntheses have been applied for plant lignans burseran (**3**) and dehydroxycubebin (**4**).

## 2. Discussion on the Synthesis of Tetrahydrofuran Lignans

### 2.1. Building the Lignan Skeleton to a Furan or a Tetrahydrofuran

**Scheme 1:**
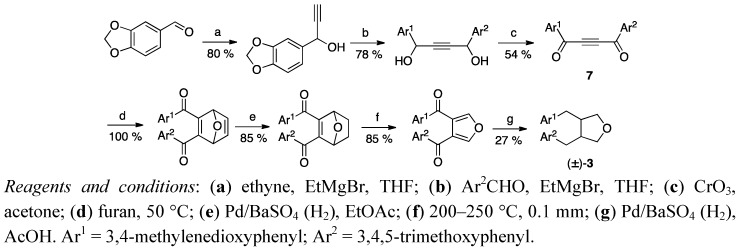
The first synthesis of burseran (**3**).

In this review the various ways of synthesising the 3,4-dibenzyltetrahydrofuran lignans are divided according to whether the lignan skeleton is built by introducing appropriate substituents directly to a furan, a 2,5-dihydro- or a tetrahydrofuran ring, or whether the formation of the tetrahydrofuran ring is the last stage in a multistep synthesis. The first synthesis of burseran (**3**) may be counted as one of the former approaches, though it is a unique method utilising the Diels-Alder reaction of a furan and acetylenic dione **7** to construct the tetrahydrofuran framework (Scheme 1). Selective hydrogenation and a retro-Diels-Alder pyrolysis led to a mixture of *cis* and *trans* isomers of racemic burseran (**3**) [23].

**Scheme 2:**
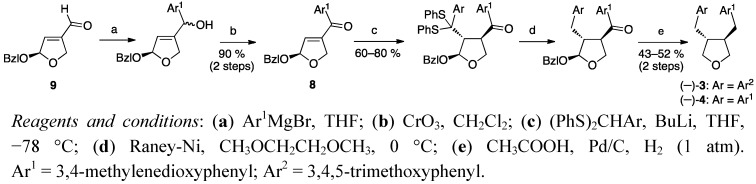
Enantiopure lignans by conjugate addition of dithioacetals to chiral dihydrofuran ketones.

Rehnberg’s and Magnusson’s approach involved the diastereospecific Michael addition of a dithioacetal to a chiral dihydrofuran ketone (**8**, Scheme 2) as the key reaction step [24]. 

The benzyloxy-substituted starting material **9** is prepared in 15%–20% overall yield over six reaction steps, starting with D- (and L-)-arabinose. The 3,4-*trans-*stereochemistry is established during the Michael addition, when the benzyloxy group forces the nucleophile to an anti-attack, and the steric bulk of the aryldithiane moiety in turn forces the aryl ketone moiety into a *trans* relationship. The desulfurized products were submitted to hydrogenolysis over palladium on carbon, to yield variously oxygenated tetrahydrofurans depending on the reaction conditions used. The phenyl ketone moiety was reduced to a benzylic group in both acidic and basic media. When reductions were performed in acidic media, the fully reduced tetrahydrofurans burseran (**3**) and dehydroxycubebin (**4**) were obtained, whereas in neutral or basic media the hemiacetals trichostin and cubebin were the products.

**Scheme 3:**
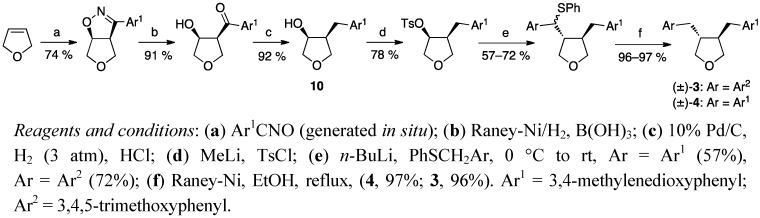
Intermolecular nitrile oxide cycloaddition to 2,5-dihydrofuran.

Gaboury and Sibi’s synthesis of racemic [25] or enantiopure [26] tetrahydrofuran lignans begins with the cycloaddition of a nitrile oxide to 2,5-dihydrofuran (Scheme 3). After reduction and tosylation, the other benzyl substituent is introduced to the furan ring via an S_N_2 displacement. A lipase-mediated kinetic resolution of the alcoholic intermediates **10** furnished both enantiomers of the lignan precursors in high optical purity and enabled the subsequent synthesis of pure enantiomers [26].

**Scheme 4:**
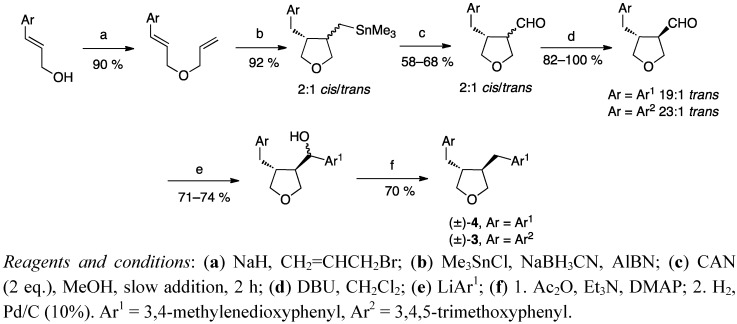
Synthesis of burseran (**3**) and dehydroxycubebin (**4**) via radical mediated carbocyclisation.

Another approach to synthesise tetrahydrofuran lignans was reported by Hanessian and Léger [27]. Radical-mediated carbocyclisation of a diene, obtained from a substituted cinnamyl alcohol, gave a tetrahydrofuran derivative (Scheme 4). Destannylation and epimerization of the aldehyde intermediate with DBU gave the *trans* enriched isomer. Further reactions on this aldehyde furnished racemic burseran (**3**) and dehydroxycubebin (**4**). Esterification of the preceding alcohols with (*S*)-*O*-methyl mandelic acid gave the corresponding esters which were separable by column chromatography into their diastereomers and hydrolysed to pure enantiomers.

**Scheme 5:**
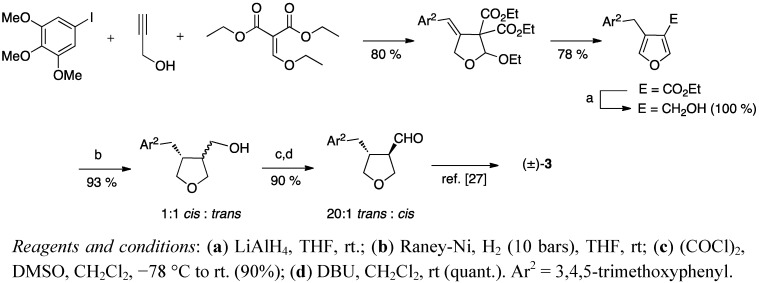
Synthesis of burseran (**3**) via 3-benzylfuran.

As a part of developing new strategies for the synthesis of heterocyclic structures, Garçon *et al.* reported a one-pot synthesis of 4-benzyl-3-carboxylate furans and their use as tetrahydrofuran lignan precursors (Scheme 5) [28]. The lignan skeleton was finally achieved with Hanessian’s and Léger’s procedure [27].

### 2.2. Cyclisation of a 1,4-Butanediol to a Tetrahydrofuran

Derivatising is a useful way to study and characterise natural products. Several groups have converted a lactone lignan natural product to a tetrahydrofuran lignan. The lactone is reduced to a 1,4-butanediol, in most cases with lithium aluminum hydride, followed by dehydration to the tetrahydrofuran lignan. Dehydration is usually carried out either under acidic conditions or by elimination of a tosylate in pyridine.

Haworth and coworkers used potassium hydrogen sulfate to convert substituted 1,4-butanediols to tetrahydrofuran derivatives. The diol was heated with potassium hydrogen sulfate at 180 °C for 0.5 h [29,30]. Eich *et al*. isolated the *trans*-dibenzylbutyrolactone type lignans (−)-matairesinol and (−)-arctigenin from a *Forsythia* species, converted the lactone to the corresponding diols with LiBH_4_ and further to tetrahydrofurans by treatment with *p*-toluenesulfonic acid in dichloromethane [31].

**Scheme 6:**
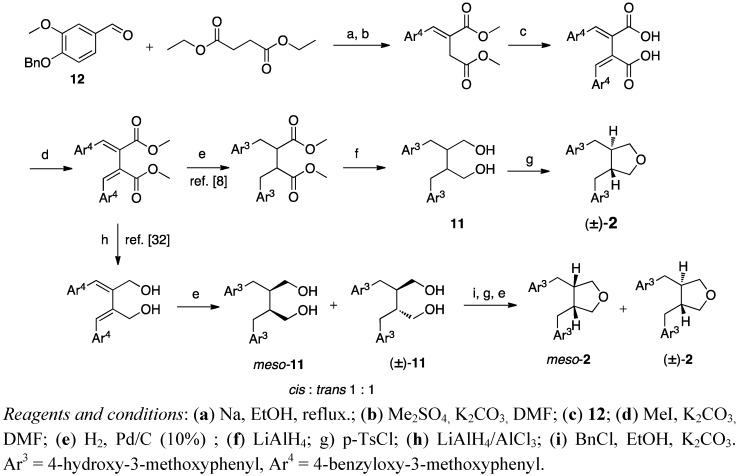
Synthesis of the lignan skeleton via Stobbe condensation and standard reduction.

Structure elucidation of a natural product lignan was performed by synthesising anhydrosecoisolariciresinol (**2**) via the Stobbe procedure [8]. The diol secoisolariciresinol (**11**) (Scheme 6), formed after condensations, hydrogenation, debenzylation and reduction, was treated with *p*-tosyl chloride to give the tetrahydrofuran lignan anhydrosecoisolariciresinol (**2**). Matsuura *et al*. reported no other products than the racemic diol **11** and further the tetrahydrofuran (±)-**2** [8]. In contrast, when Xia *et al*. exploited the Stobbe procedure in their total synthesis of ferulate derivatised anhydrosecoisolariciresinol, they performed the hydrogenation and deprotection steps in a different order and achieved a 1:1 mixture of *meso*- and (±)-secoisolariciresinol (**11**) (Scheme 6) [32].

**Scheme 7:**
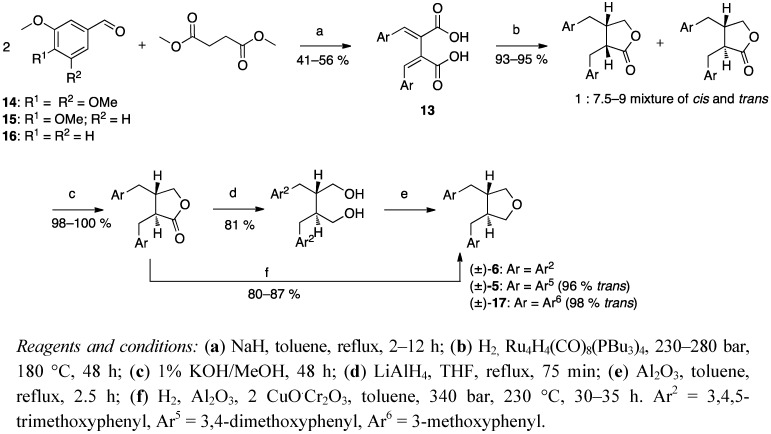
Stobbe condensation, and a one step autoclave procedure from lignano-9,9′-lactones to corresponding 9,9′-epoxylignans.

Stobbe condensation was also the first step in the procedure of Coran *et al*. [33], but they used two equivalents of the starting material to get a fulgenic acid **13** (Scheme 7). Catalytic hydrogenation of **13** yielded a mixture of *trans*- and *cis*-dibenzylbutyrolactone lignans, which was *trans* enriched in methanolic potassium hydroxide solution [34]. Their first approach to a tetrahydrofuran structure was via the corresponding diol, from the usual LiAlH_4_ reduction, which was dehydrated with Al_2_O_3_ to cause ring closure to the tetrahydrofuran. However, they improved the route with an autoclave procedure, where the lactones were hydrogenated with a copper chromite catalyst to the diols, which were concomitantly dehydrated to tetrahydrofurans in one step (f in Scheme 7) [33].

**Scheme 8:**
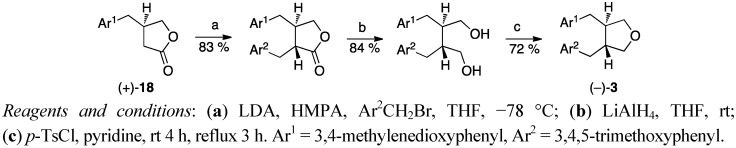
Stereoselective synthesis of burseran (**3**) from a chiral γ-butyrolactone.

The stereoselective total synthesis of optically active *trans*- and *cis*-burserans by Tomioka *et al*. started with the alkylation of a chiral piperonyl substituted γ-butyrolactone **18**. The formed lignano-9,9′-lactones were reduced to corresponding diols [35,36]. The synthesis of (−)-*trans-*burseran (**3**) is shown in Scheme 8.

**Scheme 9:**
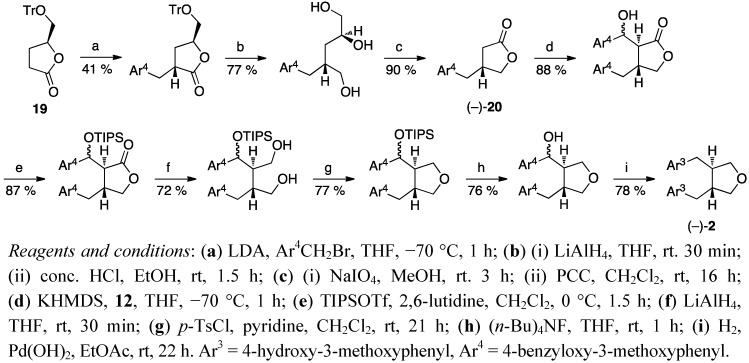
Synthesis of enantiopure anhydrosecoisolariciresinol (**2**) via hydroxybenzyl derivatives.

**Scheme 10:**
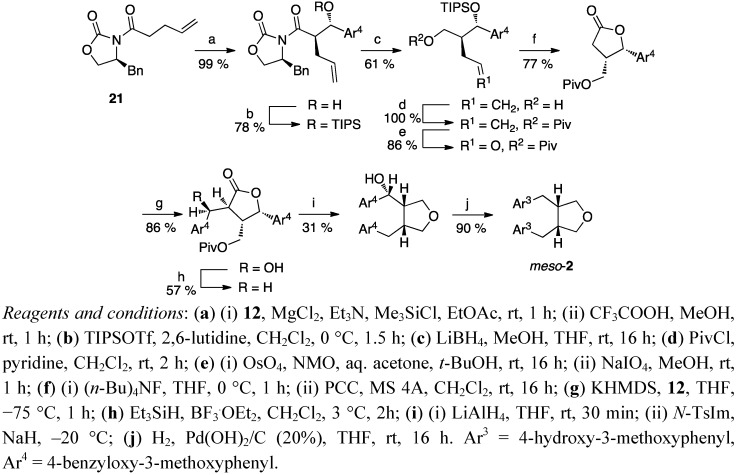
Synthesis of *meso*-anhydrosecoisolariciresinol (**2**) from an *N*-acylated oxazolidinone.

The work of Tomioka *et al*. [35,37] was used as a starting point also in the synthesis of optically active anhydrosecoisolariciresinol ((−)-**2**) by Yamauchi *et al*. [9] (Scheme 9). An optically active starting material, the γ-butyrolactone **19** was synthesised from L-glutamic acid [37], and benzylated on the α-position. The lactone was reduced, and the corresponding diol was subjected to cleavage of the trityl ether under acidic conditions to give a triol. The glycol part was oxidatively cleaved, and a new lactone ring was formed via PCC oxidation. Tomioka *et al*. alkylated the benzylsubstituted lactone (+)-**18** with benzyl bromide (Scheme 8), while Yamauchi *et al*. used benzaldehyde **12** for their analog (−)-**20** (Scheme 9). This caused extra steps to the total synthesis of anhydrosecoisolariciresinol ((−)-**2**), but enabled an access to various hydroxybenzyl derivatives [9]. The enantiomer (+)-**2** was synthesised analogously starting with D-glutamic acid, and also the synthesis of the *meso*-isomer of anhydrosecoisolariciresinol (*meso*-**2**) starting from the acylated oxazolidinone **21** (Scheme 10) was reported [11].

**Scheme 11:**
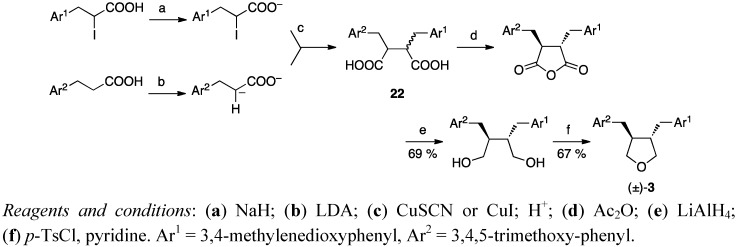
Racemic burseran (**3**) by dianion coupling.

Dianion coupling was the starting point when Belletire *et al*. synthesised racemic burseran ((±)-**3**). Reaction of the dianion of hydrocinnamic acid with an iodo-substituted carboxylate in the presence of a copper(I) salt forms a succinic acid **22**. The mixture of diacid diastereomers **22** (Scheme 11) was heated at reflux with a large excess of Ac_2_O, which smoothly epimerized the substituted benzyl chains into the thermodynamically more stable *trans* isomer with concomitant cyclisation to an anhydride, which was reduced to a diol [38]. In Tomioka’s *et al*. [35,36], Yamauchi’s *et al*. [9] and Belletire’s *et al*. [38] approaches, the 1,4-butanediols formed were cyclised to tetrahydrofurans by stirring with *p*-toluenesulfonyl chloride in pyridine.

**Scheme 12:**
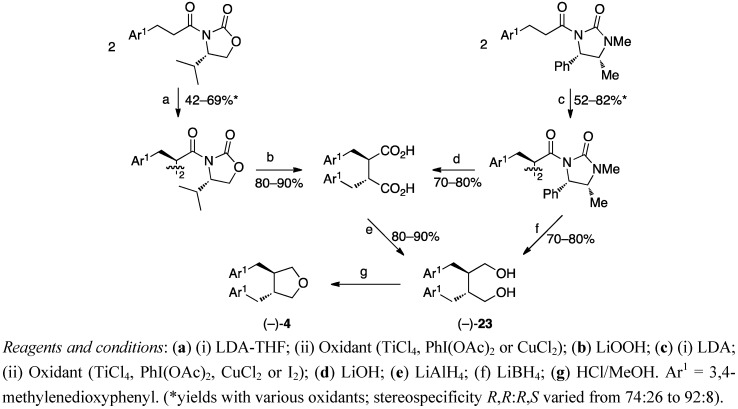
Asymmetric synthesis of dehydroxycubebin (4) via the oxidative homocoupling of a chiral hydrocinnamic acid derivative.

In the most recent studies the conversion of 1,4-butanediols to tetrahydrofuran lignans is made by refluxing in methanolic HCl for several hours [39,40,41,42]. Kise *et al*. obtained the lignan skeleton by oxidative homocoupling of optically active hydrocinnamic acid derivatives to a mixture of *R*,*R*- and *R*,*S*-dimers, which were separated by column chromatography and hydrolyzed to give dibenzylsuccinic acids and further reduced to *trans*- and *cis*-dibenzylbutanediols (from *R*,*R*- and *R*,*S*-dimers, respectively). *The trans*-dibenzylbutanediol (−)-dihydrocubebin (**23**) (Scheme 12) obtained was transformed to (−)-dehydroxycubebin (**4**) by the usual procedure of refluxing in methanolic HCl [41].

A regioselective oxidative coupling was also a key step in the procedure of Wang *et al*. [42]. The β–β coupling product was hydrogenated to give a mixture of *cis*- and *trans*- isomers (in the ratio 3:2). 

**Scheme 13:**
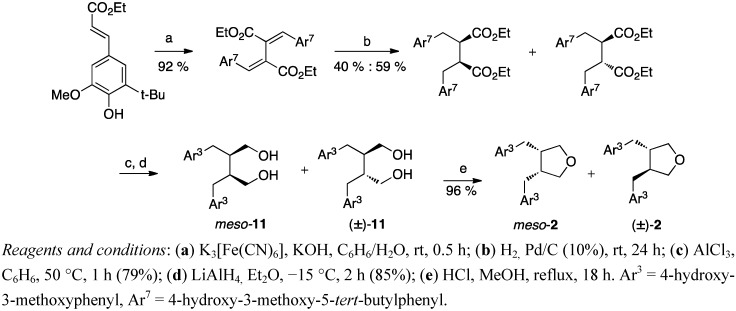
*Meso*- and racemic anhydrosecoisolariciresinols **2** via a regioselective oxidative coupling.

Cleavage of the *tert*-butyl groups and reduction with LiAlH_4_ afforded *meso*- and (±)-secoisolariciresinols **11** (Scheme 13) which were refluxed under acidic conditions to give the tetrahydrofurans *meso*- and (±)-anhydrosecoisolariciresinol (**2**), respectively.

**Scheme 14:**
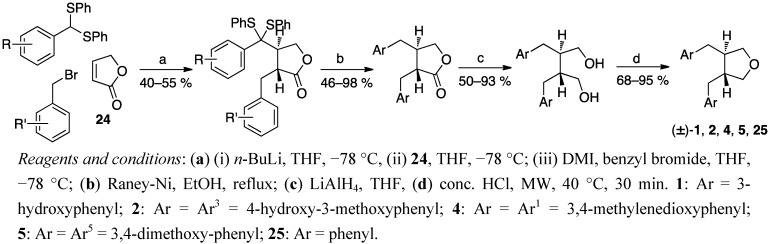
One-pot Michael addition–alkylation procedure as the key step in obtaining various lignan skeletons.

In our laboratory we have been studying a versatile route to synthesise the lignan skeleton from readily available starting materials (Scheme 14). The dibenzylbutyrolactone skeleton is prepared by a tandem Michael addition–alkylation procedure [43]. The protected dithioacetals are obtained by reacting the anion of diphenylthioacetal with 2-butenolide **24** at a low temperature, and the intermediates are then alkylated *in situ* with benzylic bromides. The substituents in the starting materials, diphenythioacetals and benzylic bromides, may be different or the same, and free phenols must be benzyl protected. Raney nickel treatment provides both desulfurisation and simultaneous removal of the benzylic protecting groups. The route is flexible in controlling the relative and absolute stereochemistry and the nature of the aryl substituents. The approach in scheme 14 gives a racemic mixture of *trans* substituted lignano-9,9′-lactones. In order to obtain optically pure products the protocol can be adjusted and controlled by a chiral Michael acceptor instead of butenolide **24** [44].

The dibenzylbutyrolactones are further reduced to diols with LiAlH_4_. The cyclisation of *trans*-dibenzyl-1,4-butanediols to *trans*-3,4-dibenzyltetrahydrofurans is performed with conc. HCl under microwave heating in 30 min [45]. Utilising this procedure and the fast and easy cyclisation reaction, we have synthesised racemic enterofuran (**1**), anhydrosecoisolariciresinol (**2**), dehydroxycubebin (**4**) brassilignan (**5**), and 9,9′-epoxylignane **25**, a tetrahydrofuran lignan framework without any substituents on the aromatic rings. In addition, we have promising preliminary results showing that this cyclisation method works well also for synthesising the furofuran structures from tetraols.

## 3. Feasible Experimental Approaches

Despite their biological activities the 3,4-dibenzyltetrahydrofuran type lignans have not been given much attention. This review compiles reported strategies for the synthesis of 3,4-dibenzyltetrahydrofuran lignans, biologically active plant and mammalian products. They are all total syntheses with different key step strategies, including Diels–Alder, Michael additions, alkylations, nitrile oxide cycloadditions, radical cyclisations, dianion or oxidative couplings. Most of the procedures were used for racemic products. Different approaches are analysed based on whether the lignan skeleton is built onto a tetrahydrofuran ring or whether the formation of the tetrahydrofuran ring is the last stage in a multistep synthesis. In the latter cases the final tetrahydrofuran lignan product is formed from a corresponding lignanodiol and often also from lignanolactone, which makes it a versatile approach to various lignan structures as needed.

The procedures that give enantiomerically pure products, Rehnberg’s and Magnusson’s (Scheme 2), Tomioka’s *et al*. (Scheme 8), Yamauchi’s *et al*. (Scheme 9) and Kise’s *et al*. (Scheme 12), start with chiral starting materials. Rehnberg and Magnusson (Scheme 2), Gaboury and Sibi (Scheme 3), Belletire *et al*. (Scheme 11), and Wähälä *et al*. (Scheme 14) have diastereomeric control over the reactions achieving only *trans*-products. Kise *et al*. affect the *cis*/*trans*-ratio with changing oxidants, while Tomioka *et al*. and Yamauchi *et al*. have separate routes for both pure diastereomers.

Although 3,4-dibenzyltetrahydrofuran lignans are rather simple natural products with only two chiral centers, the overall yields of these total syntheses range from moderate (59%) to poor (<6%) especially when the entire starting material synthesis is taken into account. Thus no procedure seems to clearly stand out above others. The overall yield is highest (59%) in the regioselective coupling of Wang *et al*. (Scheme 13), but the procedure gives a mixture of *cis*- and *trans*-products. Belletire *et al*. obtain racemic burseran (**3**), in 46% total yield from hydrocinnamic acid and iodo-substituted carboxylate (Scheme 11), which is a convenient procedure in that (like all the others except the oxidative homocouplings and the other Stobbe condensation), the aromatic moieties may be the same or differently substituted. The autoclave procedure of Coran *et al*. (Scheme 7) from butyrolactone lignans to tetrahydrofurans in one step is a good application to reduce the number of reaction steps and isolation of intermediates. However, there is a definite need for simpler, shorter and more step-economical approaches.

The use of dielectric microwave heating has been utilized in organic synthesis at an increasing rate since the pioneer work of Gedye [46] and Giguere [47] in 1986. In spite of having become in frequent and common use in laboratory work, microwave techniques have not been reported in the synthesis of lignans until very recently (e.g., in the synthesis of trisubstituted furanolignans [48], steganacin and steganone derivatives [49], and deuterium labelling [50,51,52]). In many cases microwave heating has been shown to dramatically reduce reaction times and enhance product purities, compared with conventional heating methods. In most of the reviewed methods the cyclisation of dibenzyl-1,4-butanediols to 3,5-dibenzyltetrahydrofurans takes several hours, whereas under microwave heating the conversion from diol to tetrahydrofuran is in most cases completed in less than 30 min [45].

## 4. Conclusions

Different procedures to synthesise both enantiopure and racemic 3,4-dibenzyltetrahydrofuran type lignans are reviewed and compared. The total syntheses include different key step strategies and enable preparation of various plant or mammalian lignans as well as distinct substitution patterns from the known natural ones. However, there is still room for improvement and for simpler and more step-economical approaches.
